# Small nuclear RNA-mediated modulation of splicing reveals a therapeutic strategy for a *TREM2* mutation and its post-transcriptional regulation

**DOI:** 10.1038/s41598-018-25204-2

**Published:** 2018-05-02

**Authors:** Motoaki Yanaizu, Kenji Sakai, Youhei Tosaki, Yoshihiro Kino, Jun-ichi Satoh

**Affiliations:** Department of Bioinformatics and Molecular Neuropathology, Meiji Pharmaceutical University, 2-522-1, Noshio, Kiyose-shi, Tokyo 204-8588 Japan

## Abstract

Loss-of-function mutations in *TREM2* cause Nasu-Hakola disease (NHD), a rare genetic disease characterized by early-onset dementia with leukoencephalopathy and bone cysts. An NHD-associated mutation, c.482 + 2 T > C, disrupts the splice donor site of intron 3 and causes aberrant skipping of exon 3, resulting in the loss of full-length TREM2 protein. Here, we examined the efficacy of artificial U1 and U7 small nuclear RNAs (snRNAs) designed to enhance exon 3 inclusion. Using mutant *TREM2* minigenes, we found that some modified U1, but not U7, snRNAs enhanced exon 3 inclusion and restored TREM2 protein expression. Unexpectedly, we found that exon 3 of wild-type *TREM2* is an alternative exon, whose skipping leads to reduced expression of the full-length protein. Indeed, TREM2 protein levels were modulated by modified snRNAs that either promoted or repressed exon 3 inclusion. The splice donor site flanking exon 3 was predicted to be weak, which may explain both the alternative splicing of exon 3 under normal conditions and complete exon skipping when the c.482 + 2 T > C mutation was present. Collectively, our snRNA-based approaches provide a potential therapeutic strategy for NHD-associated mis-splicing and novel insights into the post-transcriptional regulation of TREM2.

## Introduction

Genetic mutations at gene splice sites are a relatively common cause of human genetic diseases, accounting for over 10% of disease-causing mutations^1^. These mutations cause aberrant regulation in splicing, such as exon skipping and the activation of cryptic splice sites, and can compromise protein expression. If a mutation only affects the sequences of the splice donor or acceptor sites in introns, it can impair protein expression via aberrant splicing, while the remaining protein-coding sequence is not affected. In these cases, it is theoretically possible to cure the resulting diseases by therapeutic interventions that correct the splicing abnormalities. Modified small nuclear RNAs (snRNAs) have been used to modulate aberrant splicing caused by genetic mutations. U1 snRNA is a component of the spliceosome and recognizes splice donor sites (or 5′ splice sites) through direct base-paring. Selection of a 5′ splice site is influenced by several factors, including sequence complementarity between the splice site and U1 snRNA, local secondary structure of pre-mRNA, trans-acting proteins bound to adjacent regions, and competition with other 5′ splice sites^2^. Mutations in the 5′ splice site can be corrected by artificial U1 snRNAs modified to recognize the mutated 5′ splice sites^3–7^. U7 snRNA is involved in the 3′ processing of histone mRNA and is not inherently involved in pre-mRNA splicing. However, by changing the sequence bound by like-Sm (LSm) proteins, U7 snRNA can be converted into an artificial splicing factor that induces either inclusion or skipping of an exon, depending on the location of the target site on the transcript and presence of any additional modifications in the U7 snRNAs^8–10^. Typically, antisense oligonucleotide sequences to the target sites are included in engineered U7 snRNAs to allow for transcript-specific recognition by the U7 snRNA and/or to block functional sequences such as splicing signals, including splice sites, branch sites, and splicing enhancers or silencers.

Nasu-Hakola disease (NHD) is a rare autosomal recessive disorder characterized by early-onset dementia with leukoencephalopathy and bone cysts^11^. NHD is caused by loss-of-function mutations in either the triggering receptor expressed on myeloid cells 2 (*TREM2*) or TYRO protein kinase binding protein (*TYROBP*, also known as *DAP12*)^12,13^. Both are membrane proteins expressed in macrophages, microglia, and osteoclasts; these proteins bind to each other, thereby constituting a transmembrane signaling pathway^14–16^. *TREM2* mutations have been also detected in patients with frontotemporal dementia, who do not have overt bone abnormalities^17,18^. Recently, the R47H variant of TREM2 was identified as a genetic risk factor of Alzheimer’s disease (AD)^19,20^. TREM2 binds to apolipoproteins, including ApoE, another risk factor of AD, and is involved in the clearance of amyloid beta by microglia. The R47H variation of TREM2 is found in the immunoglobulin-like (Ig) domain and decreases the binding to its ligands^21,22^. TREM2 is thus a key protein linking dysfunctions in microglia or macrophages to the etiology of dementia.

In this study, we attempted to correct the aberrant splicing of *TREM2* caused by a splice site mutation flanking exon 3 (c.482 + 2 T > C), found in a patient with NHD^23^. This mutation causes skipping of exon 3 because of a disruption in a highly conserved GT dinucleotide at the beginning of intron 3. Because this mutation does not change the coding sequence of *TREM2*, protein function may be restored if the splicing is corrected. We examined several modified U1 and U7 snRNAs designed to promote *TREM2* exon 3 inclusion. By comparing the efficacy of several modified U1 and U7 snRNAs, we determined the best construct for correcting of exon 3 skipping. In this study, we unexpectedly found that exon 3 of wild-type (WT) *TREM2* is an alternative exon whose inclusion or skipping directly influences the level of TREM2 protein, suggesting a novel regulatory mechanism for this protein.

## Results

### NHD-associated splice site mutation disrupts exon 3 splicing

**Figure 1:**
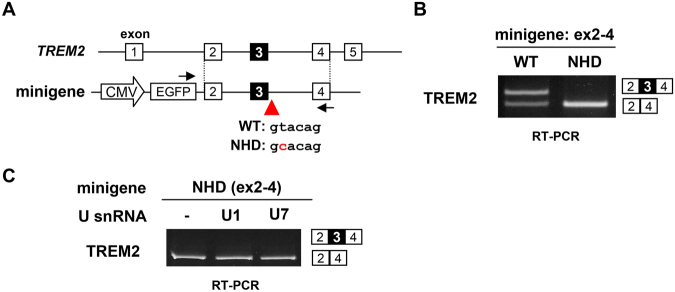
NHD minigene shows exon 3 skipping. (**A**) Schematic diagram of the *TREM2* exon 2–4 minigene. The minigene is inserted downstream of the EGFP cDNA. The positions of the primer set used to detect splicing are indicated by arrows. A red arrowhead indicates the position of the 5′ splice site mutation (c.482 + 2 T > C) found in NHD. (**B**) Splicing assay of WT and NHD minigenes transfected into HEK cells. Splicing patterns were detected by RT-PCR using the primer set indicated in (**A**). (**C**) Unmodified U1 and U7 snRNAs did not alter the splicing pattern of the NHD minigene. The NHD minigene was transfected with either an empty vector, unmodified U1 snRNA, or unmodified U7 snRNA. Splicing patters were detected as indicated in (**B**). Exon 3 inclusion was not induced by co-expression of U1 or U7 snRNA. Original gel images are shown in Supplementary Fig. S8.

We first engineered minigenes covering a genomic region from exons 2 to 4 of *TREM2* with and without an NHD-associated mutation (c.482 + 2 T > C) at the 5′ splice site of intron 3 [denoted as NHD(ex2-4) and WT(ex2-4), respectively] (Fig. 1A). These minigenes were transfected into HEK293 cells and splicing patterns were detected by reverse transcription polymerase chain reaction (RT-PCR). As expected, cells transfected with the WT minigene presented a splice product containing exon 3 (Fig. 1A). Unexpectedly, exon 3 was partially skipped in this minigene. In contrast, the NHD minigene showed complete skipping of exon 3 (Fig. 1B). Hereafter, we used the NHD minigene to examine whether engineered small RNAs could modulate the aberrant splicing of this minigene. As negative controls, we confirmed that overexpression of unmodified U1 and U7 snRNAs did not alter the splicing pattern of the NHD(ex2-4) minigene (Fig. 1C).

### Modified U1 snRNA partially corrected aberrant splicing of exon 3

**Figure 2:**
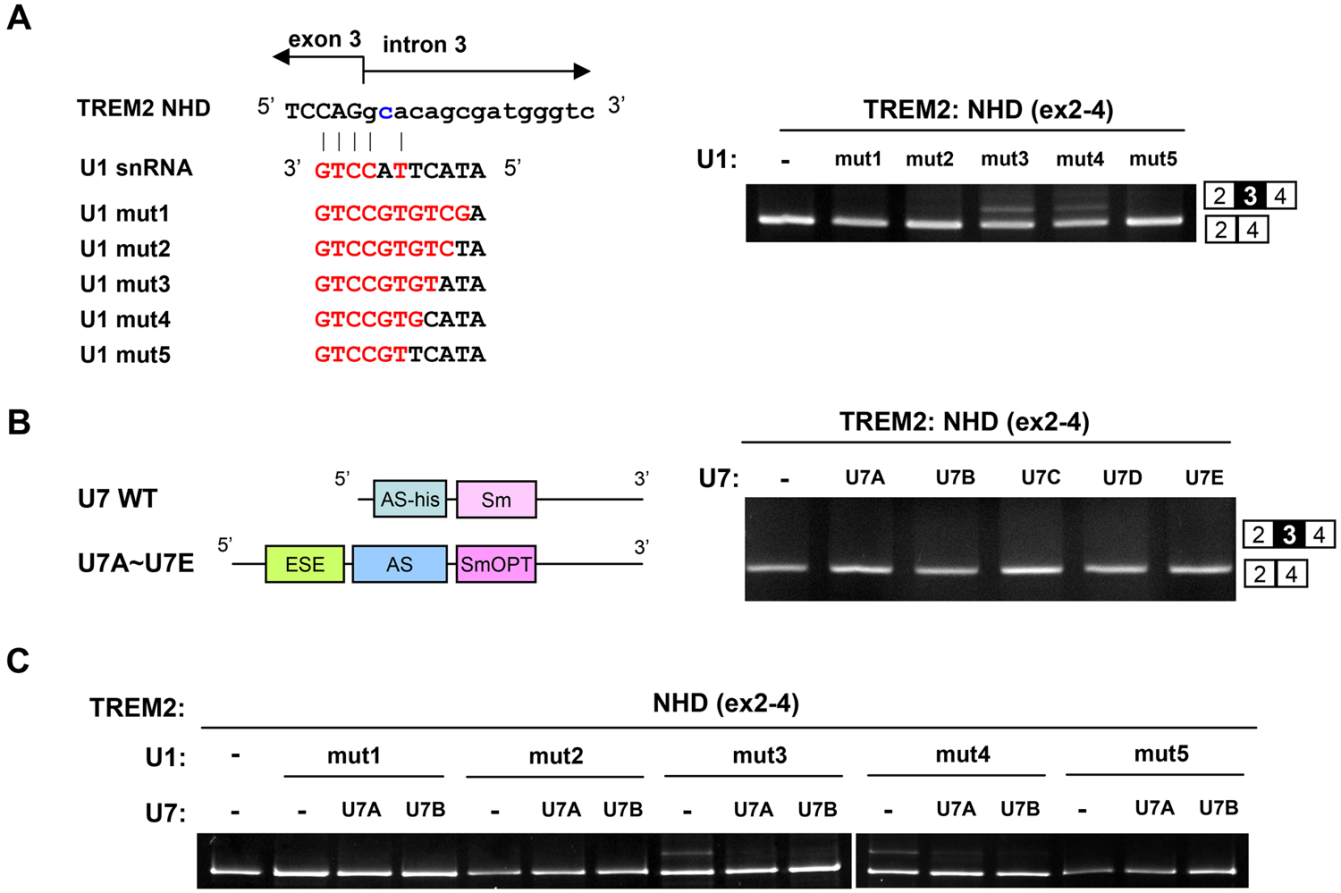
Correction of exon 3 skipping by modified U1 and U7 snRNAs. (**A**) The left panel shows the sequence of the 5′ region of the unmodified (WT) and modified U1 snRNAs used in this study. The mutated nucleotide in the NHD minigene is shown in blue. Nucleotides of the U1 snRNA constructs complementary to the 5′ splice site region of *TREM2* intron 3 are indicated in red. The right panel shows the results of a splicing assay using modified U1 snRNA. The NHD minigene was transfected with either an empty vector or modified constructs. Splicing patterns were detected by RT-PCR and agarose gel electrophoresis. U1mut3 increased exon 3 inclusion. (**B**) The left panel shows a schematic diagram of the modified U7 snRNA used in this study. U7A–U7E were designed to hybridize to different regions in exon 3 of *TREM2*. Refer to the text for the details regarding SmOPT and ESE (exonic splicing enhancer). The right panel shows the results of a splicing assay using the modified U7 snRNA. The NHD minigene was transfected with either an empty vector or modified constructs. Splicing patterns were detected as in (**A**). U7A and U7B did not increase exon 3 inclusion when used separately or simultaneously. (**C**) Co-expression of U1 and U7 constructs to modulate exon 3 splicing. Cells were transfected with the NHD minigene together with the U1 and U7 constructs as indicated. Splicing patterns were detected as in (**A**). Original gel images are shown in Supplementary Fig. S8.

We made a series of modified U1 snRNA constructs, with different complementarities to the 5′ splice site of *TREM2* intron 3 with the c.482 + 2 T > C mutation (U1mut1–U1mut5; Fig. 2A, left). When these constructs were transfected with the NHD(ex2-4) minigene, we observed partial inclusion of exon 3 in cells transfected with U1mut3 or U1mut4, with a higher efficacy for U1mut3 (Fig. 2A, right). The other constructs did not induce exon 3 inclusion.

We then created modified U7 constructs (U7A and U7B; Fig. 2B). According to previous reports, we introduced an smOPT sequence (Fig. 2B, left and Supplementary Fig. S1A) that recruits a series of LSm proteins involved in pre-mRNA splicing in place of the LSm proteins involved in histone mRNA processing^9,24^. We included a tandem array of an exonic splicing enhancer (ESE) sequence to recruit serine/arginine-rich (SR) proteins to facilitate the recognition of an exon and promote its splicing (Supplementary Fig. S1B). Among the several known ESEs, we used a GAA-rich ESE that binds to SRSF1 (formerly known as SF2/ASF) and has been previously used for splicing correction^9^. We also included an antisense sequence complementary to five different regions of exon 3 (U7A–U7E in Fig. 2B and Supplementary Fig. S1B,C). When these modified U7 constructs were transfected with the NHD(ex2-4) minigene, no inclusion of exon 3 was observed (Fig. 2B), indicating that the U7 constructs were not effective when treated alone. We then examined the combinatorial expression of both the U1 and U7 constructs. Co-expression of U1mut1 and either U7A or U7B did not enhance exon 3 inclusion (Fig. 2C). While U1mut3 alone induced exon 3 inclusion (Fig. 2A), co-expression of the U1mut3 and U7 constructs resulted in less efficient exon 3 inclusion (Fig. 2C). Thus, for U1mut3, co-expression with U7 constructs negatively modified the effect of U1mut3. In summary, U1mut3 treatment was the most effective in promoting exon 3 inclusion of the NHD(ex2-4) minigene.

### Small RNA-mediated splicing correction restored TREM2 protein expression

**Figure 3:**
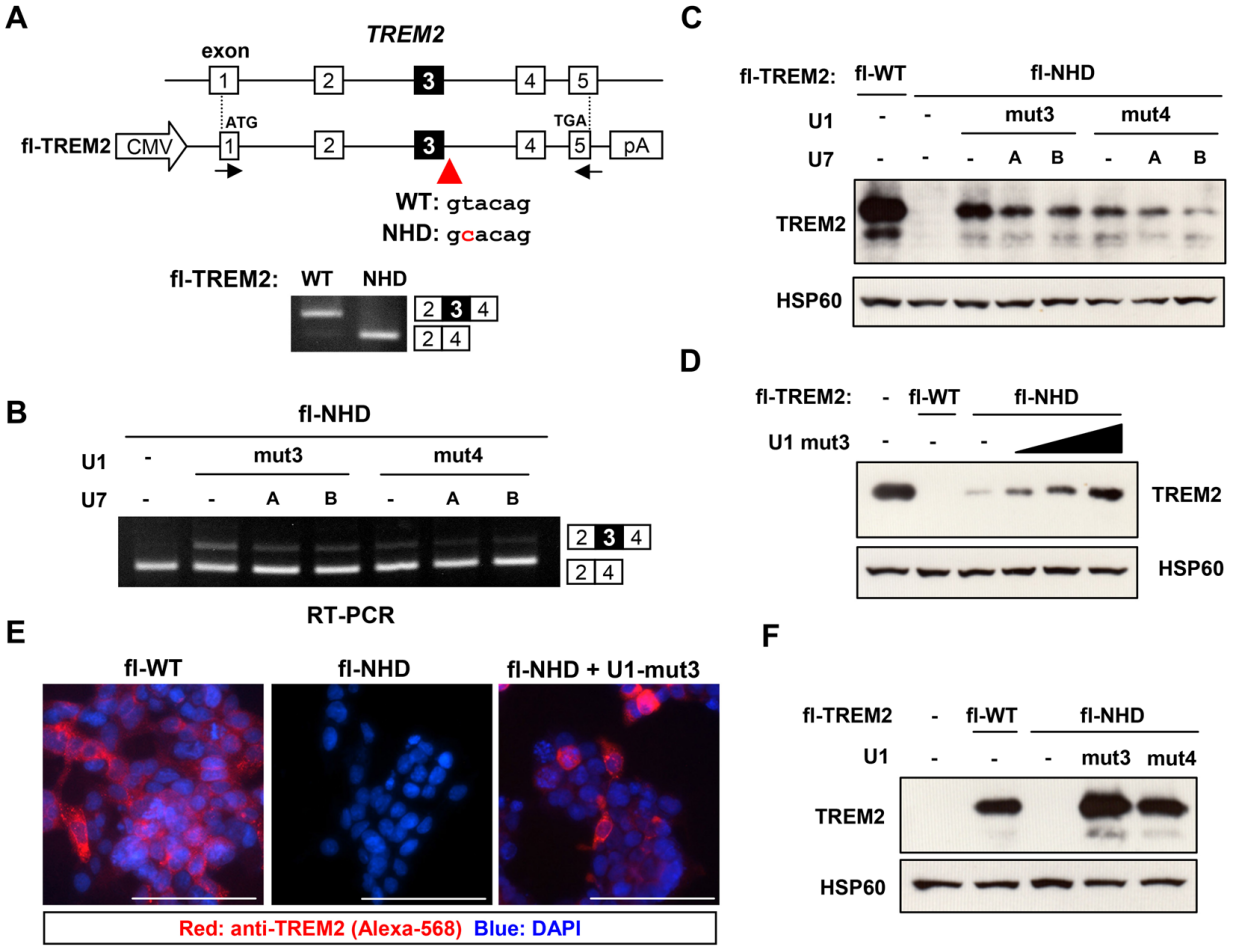
Modified U1 snRNA-mediated correction of TREM2 mis-splicing associated with NHD. (**A**) Structure of the fl-WT and fl-NHD minigenes integrated into the genome of HEK293 cells using the Flp-In system. Congenic cell lines stably harboring either minigene were established. The splicing pattern of *TREM*2 in these cell lines are shown at the bottom. (**B**) *TREM2* splicing patterns of the fl-NHD cells. For fl-NHD cells, transfection of U1mut3 induced the spliced product containing exon 3. (**C**) Western blot analysis of fl-WT and fl-NHD cells and parental HEK cells. Either U1mut3 or U1mut4 were co-transfected with U7A and/or U7B into fl-NHD cells. HSP60 was used as a loading control. (**D**) U1mut3 dose-dependently induced TREM2 protein expression in fl-NHD cells. Increasing amounts of U1mut3 were transfected into fl-NHD cells and TREM2 expression was detected by western blotting. (**E**) Immunofluorescence of fl-WT and fl-NHD cells using an anti-TREM2 antibody (red). U1mut3 induced TREM2 protein expression in fl-NHD cells. Nuclei were stained with DAPI. Scale bar: 100 μm. (**F**) Transient co-expression of U1mut3 and fl-NHD restored TREM2 protein expression. HEK cells transfected with the indicated constructs were analyzed by western blotting. Original gel images and western blot data are shown in Supplementary Fig. S8.

We then constructed minigenes covering the full protein-coding region of *TREM2* with or without the splice site mutation (fl-WT and fl-NHD; Fig. 3A,B) to evaluate the effect of splicing correction on TREM2 protein expression. We established HEK cell lines stably expressing either fl-WT or fl-NHD using the Flp-In system. First, we analyzed the basal splicing patterns of these cell lines. As expected, we detected no exon 3 inclusion in fl-NHD cells (Fig. 3A). Consistent with the above results, transcripts containing exon 3 were detected when these cells were transfected with U1mut3 and, to a lesser extent, U1mut4 (Fig. 3B). Next, we examined protein expression in both fl-WT and fl-NHD cells. TREM2 expression was detected in fl-WT cells but not in fl-NHD cells and their parental HEK cells using an anti-TREM2 antibody (Fig. 3C). When U1mut3 was transfected into fl-NHD cells, we observed TREM2 protein expression, consistent with the partial restoration of exon 3 inclusion (Fig. 3C). We also tested full combinations of U1 and U7 constructs but detected no cooperative increase in exon 3 inclusion (Fig. 3C and Supplementary Fig. S3). U1mut3 increased TREM2 protein levels in a dose-dependent manner (Fig. 3D). TREM2 expression was examined by immunofluorescence using an anti-TREM2 antibody. TREM2 protein was detected in untreated fl-WT cells, but not in untreated fl-NHD cells (Fig. 3E, left and middle panels). However, overexpression of U1mut3 in fl-NHD cells induced TREM2 protein expression in some transfected cells (Fig. 3E, right panel). As we used cell lines that can stably express a minigene, the effect of U1mut3 was likely partially masked by pre-existing *TREM2* transcripts lacking exon 3 that had been expressed prior to U1mut3 transfection. In addition, the restoration of TREM2 protein may be underestimated because some cells did not express U1mut3 and thus, TREM2 splicing was not corrected in these cells, as suggested by the results shown in Fig. 3E. We then examined transient co-expression of the fl-NHD minigene and U1 constructs in HEK cells. Remarkably, TREM2 expression was restored to a level comparable to that of the fl-WT minigene (Fig. 3F). We also tested two different cell lines, COS-7 and Neuro2a. When the fl-NHD minigene was transfected with U1mut3, TREM2 expression was restored, as observed in HEK cells, indicating that U1mut3 was effective in multiple cell types (Supplementary Fig. S2).

### *TREM2* exon 3 is an alternative exon flanked by a suboptimal 5′ splice site

**Figure 4:**
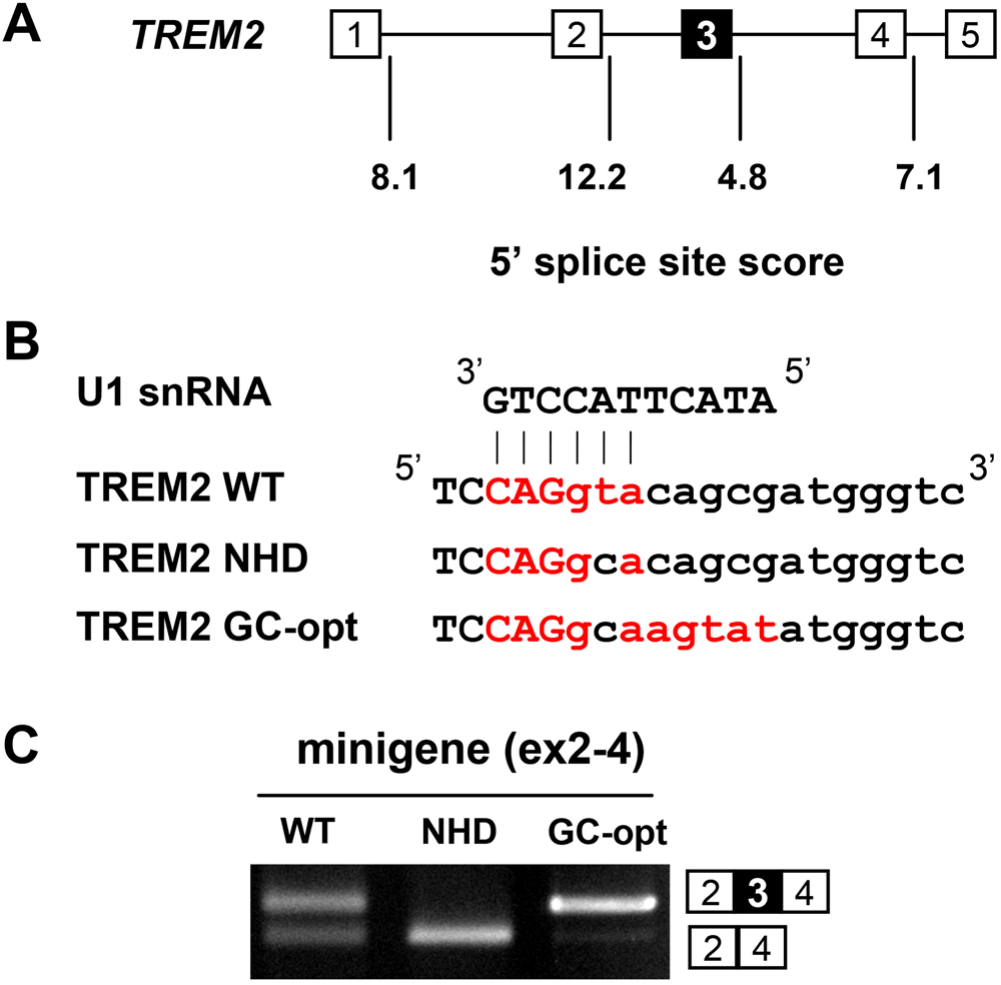
Exon 3 of *TREM2* is flanked by a weak 5′ splice site that predisposes it to exon skipping. (**A**) Splice site scores of *TREM2* 5′ splice sites calculated using an online tool (see Methods). The 5′ splice site of exon 3 showed the lowest score. (**B**) Sequence of the 5′ splice site flanking to TREM2 exon 3 with and without mutations. NHD contains a GT-to-GC mutation. GC-opt contains a GT-to-GC mutation but is otherwise an optimized sequence of a 5′ splice site complementary to unmodified U1 snRNA. The red letters indicate nucleotides complementary to U1 snRNA. (**C**) Splicing patterns of *TREM2* minigenes in HEK cells. The GC-opt minigene showed predominant inclusion of exon 3. Original gel images are shown in Supplementary Fig. S8.

Our data indicated that exon 3 in the WT(ex2-4) minigene was partially skipped (Fig. 1B), suggesting that exon 3 of *TREM2* is an alternative exon. In general, alternative exons are associated with suboptimal splice sites that only weakly match with the consensus sequences of splicing signals. We examined the strength of the *TREM2* 5′ splice site using an online tool (see Methods). In this scoring system, higher scores indicate stronger splice sites. An ideal 5′ splice site, AAG/gtaagt, shows the maximum score, 12.6, while the mean score of constitutive 5′ splice sites is 8.1. Remarkably, the score for the 5′ splice site flanking exon 3 was 4.8, the lowest among the *TREM2* exons (Fig. 4A). A similar analysis using another online tool (HSF3.0) also indicated that *TREM2* exon 3 is flanked by weak 5′ and 3′ splice sites (Supplementary Fig. S4). Thus, the 5′ splice site of *TREM2* exon 3 is a suboptimal splice site that may allow for alternative splicing. We constructed an additional minigene, GC-opt, containing a GT-to-GC substitution at the beginning of intron 3 but otherwise perfectly matched to the 5′ end of endogenous U1 snRNA (Fig. 4B). Remarkably, this minigene showed nearly complete inclusion of exon 3 despite the presence of a GT-to-GC substitution (Fig. 4C), suggesting that complete skipping of exon 3 in the NHD minigene reflected the intrinsic weakness of the flanking 5′ splice site. Although the substitutions introduced into the GC-opt minigene were predicted to create a 5′ splice site 4 nucleotides downstream of the original site, sequencing of the spliced product revealed that the original 5′ splice site with the GC dinucleotide was used for splicing (Supplementary Fig. S5).

### Skipping of *TREM2* exon 3 leads to reduced TREM2 protein expression

**Figure 5:**
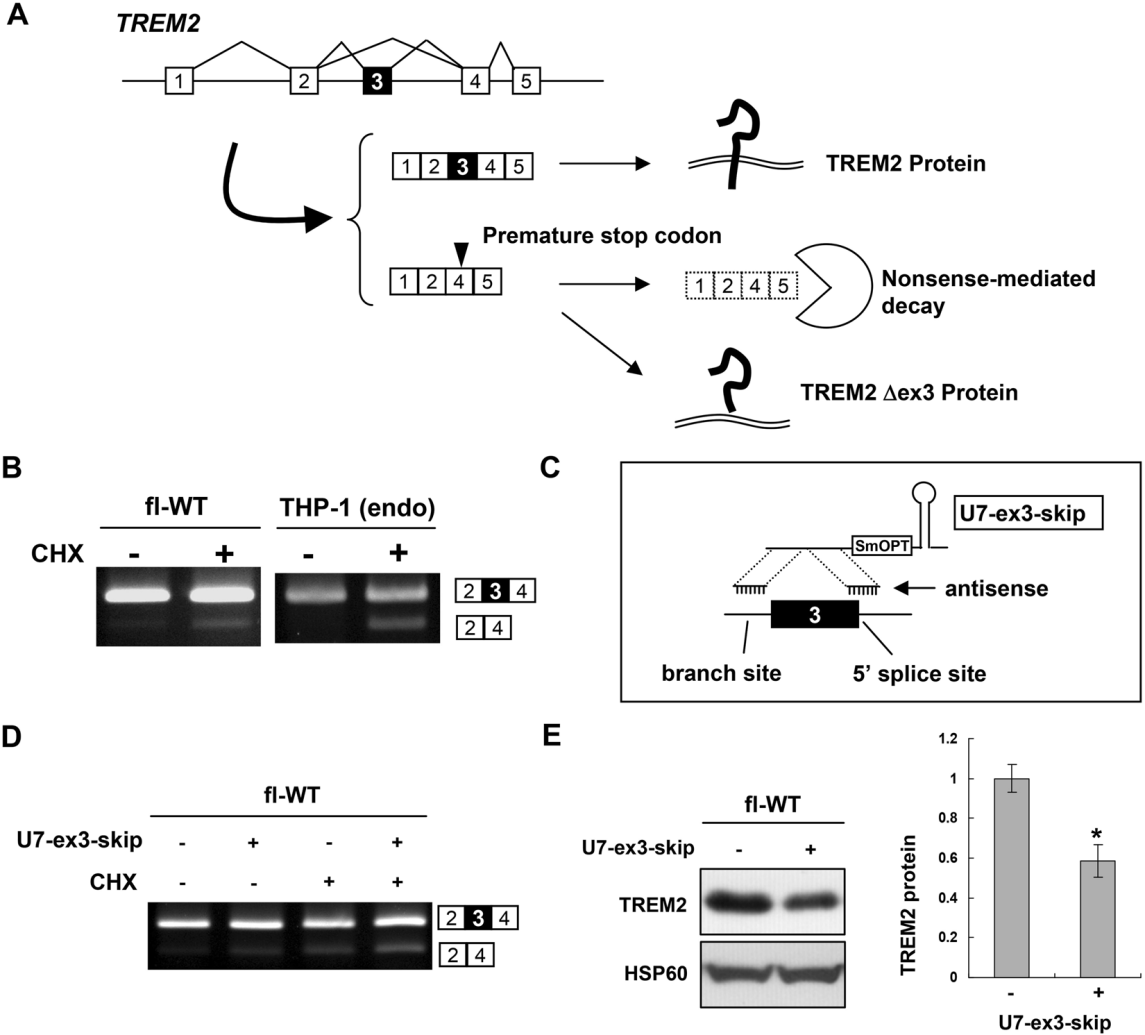
Exon 3 splicing of *TREM2* is a determinant of its protein expression. (**A**) Schematic model of *TREM2* exon 3 splicing. Exon 3 inclusion leads to expression of full-length TREM2 protein. Exon 3 skipping would result in either expression of a TREM2 isoform lacking exon 3 or degradation of mRNA via nonsense-mediated mRNA decay (NMD) due to production of a premature termination codon in exon 4. (**B**) Exon 3 is alternatively spliced in THP-1 cells. The splicing patterns of *TREM2* in fl-WT and THP-1 cells with or without cycloheximide treatment, an NMD inhibitor, are shown. (**C**) Schematic illustration of U7-ex3-skip containing antisense sequences to both the branch point of intron 2 and exon/intron junction of exon 3. (**D**) fl-TREM2 (WT) cells were treated with U7-ex3-skip and/or CHX and the splicing patterns were analyzed by RT-PCR. The exon 3-skipped pattern was increased by U7-ex3-skip and further enhanced by CHX, suggesting that some of the spliced products of exon 3 skipping were degraded by NMD. (**E**) Western blot analysis of TREM2 expression in fl-WT cells with or without transfection of U7-ex3-skip (left panel). The bar chart shows the quantification of the western blot results (mean ± SE). *P = 0.003 in a two-tailed *t*-test (n = 6). Original gel images and western blot data are shown in Supplementary Fig. S8.

**Figure 6:**
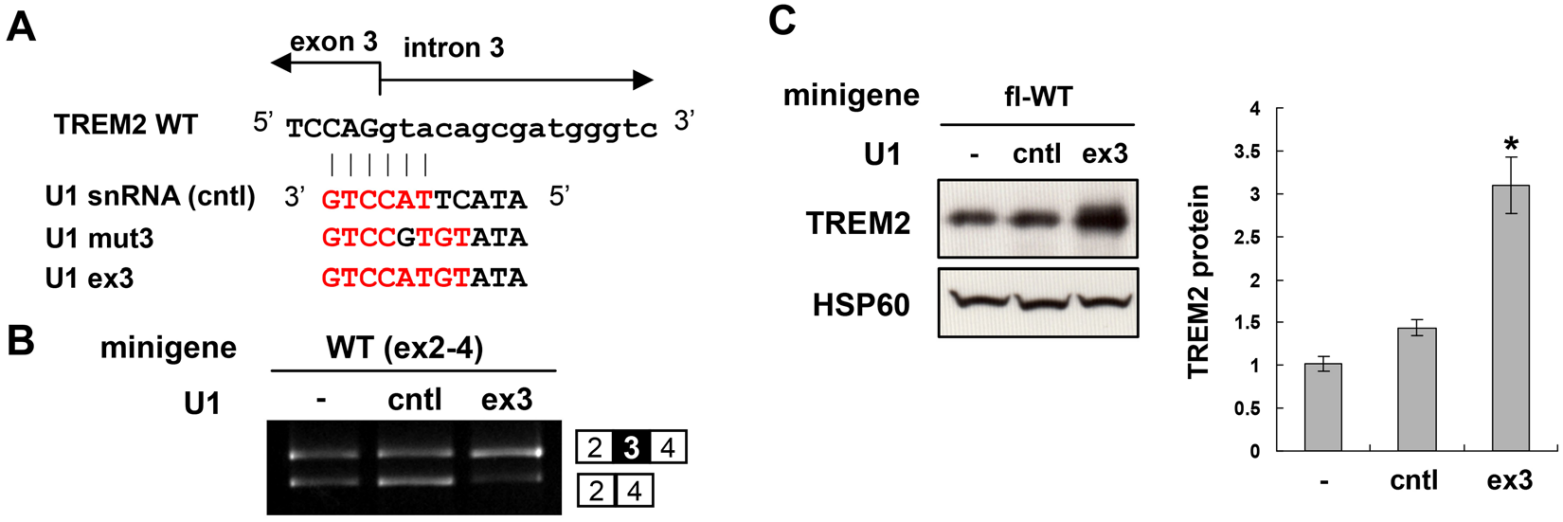
Induction of exon 3 inclusion of fl-WT minigene increased TREM2 protein expression. (**A**) Structure of the 5′ end of unmodified U1 snRNA (cntl) and modified U1 construct based on U1mut3 but matches wild-type (WT) exon 3. (**B**) Splicing assay of WT(ex2-4) minigene transfected with unmodified U1 snRNA or the U1-ex3 construct in HEK cells. (**C**) U1-ex3 increased TREM2 protein expression from the fl-WT minigene in HEK cells. Western blot analysis of TREM2 expression (left panel). Bar chart shows the quantification of the western blot results (mean ± SE, n = 4). *P = 0.00013 (-*vs*. ex3) and P = 0.0007 (cntl *vs*. ex3) in Tukey’s multiple comparison test. Original gel images and western blot data are shown in Supplementary Fig. S8.

Nonsense-mediated mRNA decay (NMD) is a mechanism that destabilizes mRNAs containing a premature termination codon (PTC), which is a stop codon located more than ~50 nucleotides upstream of the final exon-exon junction and is recognized by mRNA surveillance system during the initial round of translation^25^. Notably, exon 3 skipping in the context of the full-length *TREM2* gene was predicted to generate a PTC in exon 4, which is located >50 nucleotides upstream of the final exon-exon junction and would induce NMD, causing mRNA destabilization (Fig. 5A). In contrast, exon 3 skipping in the exon 2–4 minigene would not induce NMD, as the termination codon is located downstream of the final exon-exon junction and is therefore not recognized as a PTC. To examine whether TREM2 isoforms lacking exon 3 in fl-WT cells are suppressed by NMD, fl-WT cells were treated with cycloheximide (CHX), an inhibitor of translation that also inhibits NMD. CHX treatment increased the levels of *TREM2* transcript lacking exon 3 compared to control dimethyl sulfoxide treatment (Fig. 5B, left). Under the same conditions, we confirmed that an NMD-sensitive spliced product of *SRSF6* was increased as a positive control^26^ (Supplementary Fig. S6A). In addition, knockdown of UPF1, an essential component of NMD, increased TREM2 lacking exon 3 (Supplementary Fig. S6B). We also examined the splicing of endogenous *TREM2* in THP-1 cells. As with fl-WT cells, *TREM2* lacking exon 3 was detected by CHX treatment (Fig. 5B, right). These results suggest that (i) exon 3 skipping is a genuine pattern of endogenous *TREM2* splicing and (ii) the exon 3-skipping product is degraded by NMD. Next, we examined the effect of forced skipping of exon 3 in fl-WT cells to confirm the causal relationship between exon 3 skipping and reduced protein expression. For this purpose, we used another engineered U7 construct designed to block both the branch point and 5′ splice site associated with *TREM2* exon 3 (U7-ex3-skip, Fig. 5C). As expected, U7-ex3-skip induced partial skipping of exon 3 and reduced protein expression (Fig. 5D,E). Finally, we examined forced induction of exon 3 inclusion of the WT minigenes using a modified U1 construct (U1-ex3) that was identical to U1mut3 except that it matched the WT splice site (Fig. 6A). To confirm its effect on exon 3 splicing, we used WT(ex2-4) rather than fl-WT to avoid NMD. As expected, U1-ex3, but not unmodified U1 snRNA (cntl), increased exon 3 inclusion (Fig. 6B). When transfected with fl-WT cells, U1-ex3 significantly increased the amount of TREM2 protein (Fig. 6C). These results indicate that alternative splicing of exon 3 is a determinant of the protein expression of TREM2.

## Discussion

In this study, we examined the effects of modified U1 and U7 snRNAs on the splicing of *TREM2* minigenes with an NHD-associated mutation in cultured cells. We found that U1mut3 was the most effective in promoting exon 3 inclusion, followed by U1mut4. In contrast, U1mut1, U1mut2, and U1mut5 did not promote exon 3 inclusion. In two previous studies, exon skipping due to mutations at the second nucleotide of introns (GT to GG and GT to GC mutations) was not corrected by highly adopted U1 constructs, as was observed for our U1mut1^6,27^. In another study, a GT to TT mutation of a 5′ splice site was corrected by a modified U1 snRNA with complementarity similar to our U1mut1^3^. Thus, the optimal design of modified U1 snRNA must be determined on a gene-by-gene basis, depending on the target sequences. It is also likely that engineered U1 snRNAs strongly matched to the mutated splice site do not necessarily lead to the highest restorative effects. Previously, therapeutic molecules based on U1 or U7 snRNA were tested separately and their cooperative effects on correcting aberrant splicing caused by 5′ splice site mutations have not been examined. Although we anticipated a synergistic improvement in splicing correction when the U1 and U7 constructs were used together, no cooperative enhancement was observed. This may be because of the inability of our modified U7 constructs (U7A–U7E) to increase exon 3 inclusion. The antisense sequence appeared to function as expected because another U7 construct (U7-ex3-skip) successfully induced exon skipping (Fig. 5D). The efficacy of U7 constructs may be improved by incorporating antisense sequences of different regions of exon 3 and/or different types of ESE. U1mut3 was the most effective construct for aberrant splicing caused by the NHD mutation (Fig. 3). For further developments in gene therapy, it would be necessary to examine the effectiveness of U1mut3 in patient-derived cells and animal models. In addition, the off-target effects of U1mut3 must be investigated to evaluate the potential toxicity of this molecule. It is also important to test for an effective gene delivery system that expresses therapeutic molecules in disease-relevant cell types such as microglia, macrophages and osteoclasts. Recombinant adeno-associated viral vectors of different serotypes have been used for gene transduction into microglia and macrophages^28,29^. Selective expression of U1mut3 in the above cell types using an appropriate promoter is necessary to minimize off-target effects. While our approach was based on conventional direct base-pairing between U1 snRNA and the 5′ splice site, recent studies revealed that U1 snRNA can promote exon inclusion by binding to intronic sequences downstream of the 5′ splice site or by activating a cryptic splice site^27,30–32^. Furthermore, antisense oligonucleotides and chemical splicing modulators have been used to promote exon inclusion in therapeutic studies of spinal muscular atrophy^33–38^. These strategies should be examined in the context of our *TREM2* mutation.

**Figure 7:**
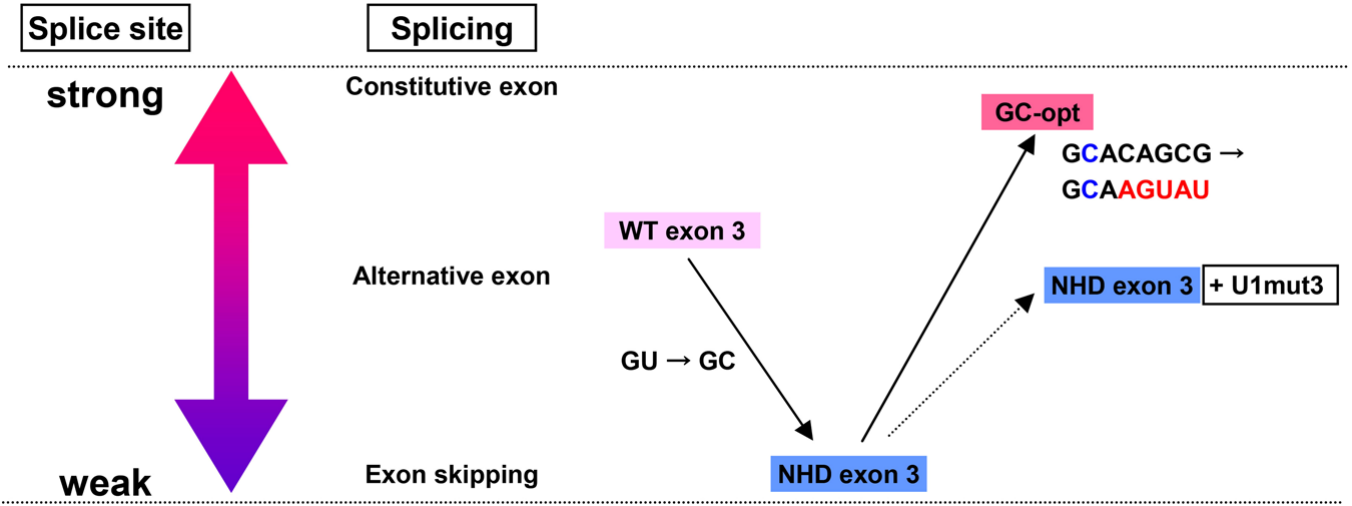
Suboptimal 5′ splice site of exon 3 underlies its alternative splicing and a predisposition to the NHD-associated mutation. Weak splicing signals are associated with alternative splicing. The 5′ splice site of *TREM2* exon 3 is a weak splicing signal, which accounts for its alternative splicing. An NHD-associated GT-to-GC mutation (c.482 + 2 T > C) caused complete skipping of this exon. However, even in the presence of the GT-to-GC mutation, the GC-opt minigene showed nearly complete inclusion of exon 3, suggesting that the GT-to-GC mutation could have less deleterious effects if exon 3 had a stronger 5′ splice site.

We found that exon 3 acts as an alternative exon that determines TREM2 protein levels. In general, weak splice sites are associated with alternative splicing. The 5′ splice site of *TREM2* exon 3 was predicted to be suboptimal (Fig. 4), consistent with our observation that exon 3 was skipped in some transcripts (Figs 1B and 5B,D). The c.482 + 2 T > C mutation further weakened the splice site, resulting in the absence of exon 3 inclusion and subsequent protein expression (Figs 1 and 3). We speculate that if *TREM2* exon 3 had a stronger consensus sequence and therefore acted as a constitutive exon, the c.482 + 2 T > C mutation would have less deleterious effects on the splicing of exon 3, as evidenced by GC-opt in Fig. 4C. Thus, our analysis suggests that the disease pathogenicity of the c.482 + 2 T > C mutation of *TREM2* reflects the weakness of the 5′ splice site of exon 3, which also explains the alternative splicing of this exon (Fig. 7). Notably, GC-AG introns are rare but naturally occurring introns in mammalian genomes, accounting for 0.5–1% of all human introns, and are associated with both constitutive and alternative splicing^39,40^. Like canonical GT-AG introns, GC-AG introns are processed by the U2-type spliceosome. These observations suggest that a GT to GC substitution alone does not inherently cause complete exon skipping as observed in our NHD minigenes. Using modified U1 snRNAs that support the recognition of the mutated exon by the spliceosome, exon 3 inclusion as well as TREM2 protein expression can be increased.

*TREM2* mRNA lacking exon 3 is predicted to be repressed by NMD. Consistently, the exon 3-skipping product was detected more easily when NMD was blocked (Fig. 5B). This susceptibility to NMD may explain why *TREM2* transcripts lacking exon 3 were unidentified. As described above, the *TREM2* exon 2-4 minigene does not elicit NMD, which led to the identification of exon 3 skipping in WT TREM2 (Fig. 1B). Moreover, using THP-1 cells, we showed that exon 3 skipping is a naturally occurring splicing pattern (Fig. 5B). In the Sequence Read Archive database, we found several RNA-seq reads supporting exon 3 skipping of *TREM2* (Supplementary Fig. S7A). In contrast to fl-WT cells, we clearly detected exon 3-skipping products in fl-NHD cells without CHX treatment. This may be because the transcript levels exceeded the capacity of NMD. Thus, *TREM2* transcripts lacking exon 3 (Δex3) are partially degraded by NMD and the remaining fraction may be translated. TREM2 Δex3 protein is predicted to contain a signal peptide at the N-terminus as well as the ligand-binding Ig domain but not the transmembrane domain (Supplementary Fig. S7B). This isoform was not detected by the anti-TREM2 antibody used in this study. Further studies are needed to characterize this isoform.

The exon 4 of *TREM2*, encoding a trans-membrane domain, is alternatively spliced and determines whether TREM2 is secreted or membrane-bound^41^. Splice site analyses indicated that the strength of the splice site of exon 3 is weaker than that of exon 4 (Fig. 4A and Supplementary Fig. S4), supporting that exon 3 is an alternative exon. Our results indicate that protein expression of TREM2 is regulated at the level of RNA processing via alternative splicing of exon 3. It is likely that some RNA-binding proteins regulate alternative splicing of *TREM2* exon 3 positively or negatively, thereby fine-tuning the expression level of TREM2 protein. Our *TREM2* minigenes may be useful for identifying such splicing regulators of *TREM2*. As TREM2 has been implicated in several diseases and conditions, including NHD, AD and frontotemporal dementia, the identification of TREM2 regulators may provide novel opportunities for therapeutic interventions in TREM2-associated diseases. Overall, starting from a specific *TREM2* mutation associated with NHD, our analysis revealed a novel post-transcriptional regulatory mechanism for TREM2, which may have broader implications for TREM2-associated disorders and their therapeutic approaches.

## Methods

### Plasmids

The primer sequences used for this study are listed in Supplementary Table S1. A human *TREM2* genomic fragment comprising exons 2 to 4 was amplified by PCR using KOD-plus-NEO (Toyobo, Tokyo, Japan) and a primer set, BclI-TREM2-Fw and SalI-TREM2-Rv. The amplified fragment was digested with *Bcl*I and *Sal*I and then inserted into the *Bgl*II-*Sal*I site of a pEGFP-C1 (Clontech, Mountain View, CA, USA). We engineered both WT and NHD minigenes, which were denoted as WT(ex2-4) and NHD(ex2-4), respectively. In these constructs, *TREM2* fragments were in-frame with the *EGFP* sequence and expressed as a part of an EGFP-fused protein. The GC-opt minigene was engineered from the WT(ex2-4) minigene by introducing an optimized 5′ splice site sequence (as shown in Fig. 5B) using a primer set, TREM2-GC-opt-Fw and TREM2-GC-opt-Rv. To create a mammalian expression vector compatible with the Flp-In system, we modified the pEF5/FRT/V5-D-TOPO plasmid (Thermo Fisher Scientific, Waltham, MA, USA) containing an insert sequence (amyloid precursor protein cDNA) at the D-TOPO site. We first replaced the EF1alpha promoter sequence with that of a CMV promoter excised from a pcDNA3.1-V5-His (Thermo Fisher Scientific) using BglII and NotI. The resulting vector was denoted as pCMV-FRT. To engineer full-length minigenes, *TREM2* genomic fragments comprising the full coding region (from the start codon in exon 1 to the stop codon of isoform 2, NP_001258750, in exon 5) were amplified by PCR with a primer set, NheI-TREM2-ex1-Fw and NotI-TREM2-ex5-Rv, and inserted into the *Nhe*I-*Not*I site of the pCMV-FRT plasmid, replacing the amyloid precursor protein cDNA.

To create artificial U1 snRNA constructs, a U1 genomic fragment including the promoter region was amplified from human genome DNA using a primer set, BamHI-U1-snRNA-Fw and XhoI-U1-snRNA-Rv, and cloned into the *Bgl*II-*Xho*I site of a pcDNA3.1-V5/His-A plasmid. Using this unmodified U1 snRNA (or cntl) as a template, mutations were introduced by PCR, as illustrated in Fig. 2A. To create the modified U7 constructs, a U7 genome fragment was amplified from mouse genome DNA using BglII-Rnu7-Fw and XhoI-Rnu7-Rv and cloned into the *Bgl*II-*Xho*I site of the pcDNA3.1-V5/His-A plasmid. We replaced the original Sm-binding sequence, AATTTGTCT, with AATTTTTGG (SmOPT), which is a consensus Sm-biding sequence from major spliceosomal snRNAs. As shown in Fig. 2B and Supplementary Fig. S1, we also replaced the histone antisense sequence with five different antisense sequences of *TREM2* (A–E) as well as an ESE, GGAGGACGGAGGACGGAGGAC, which contains sequences recognized by SRSF1 (underlined)^9^. To induce exon skipping, we removed the ESE sequence from the modified U7-A construct and replaced the antisense sequence with those of the predicted branch point of intron 2 and junction between exon 3 and intron 3 of *TREM2* (U7-skip-ex3, Fig. 5C) according to the design reported in a previous study^8^.

### Cell culture and treatments

HEK cells were maintained in Dulbecco’s Modified Eagle’s medium supplemented with 10% fetal bovine serum and penicillin/streptomycin (Thermo Fisher Scientific) at 37 °C in 5% CO_2_. THP-1 cells were maintained in RPMI 1640 medium supplemented with 10% fetal bovine serum, penicillin/streptomycin, and 1x GlutaMAX supplement (Thermo Fisher Scientific) at 37 °C in 5% CO_2_. Cells were plated in either 12- or 6-well culture plates on the day before transfection. For cells in a 12-well plate, 0.5 μg of plasmid was transfected using 1 μL of Lipofectamine 2000 (Thermo Fisher Scientific). The amount of plasmid and Lipofectamine 2000 were adjusted according to the size of the wells. For expression analyses of TREM2 protein in the presence of modified U1 or U7 constructs, G418 was added at 24 h post-transfection. The cells were then incubated for a further 72 h for enrichment. When necessary, fl-WT stable cell lines and THP-1 cells were treated with cycloheximide (Wako, Osaka, Japan), a translation inhibitor that also inhibits nonsense-mediated mRNA decay, at a final concentration of 50 μg/mL for 9 h (fl-WT) or 10 μg/mL for 3 h (THP-1). For knockdown of *UPF1*, siRNA targeting *UPF1* or firefly luciferase (Bioneer, Daejeon, Korea) was transfected into fl-WT cells (30 pmol per well of a 12-well plate) using Lipofectamine RNAiMAX (Thermo Fisher Scientific). Knockdown efficiency was confirmed by RT-PCR using primers for *UPF1* and *GAPDH* (Supplementary Table S1).

### Cellular splicing assay

A splicing assay was performed as previously described^42,43^. Cells were cultured in 12-well plates and transfected with 0.5 μg of plasmids for U1/U7 snRNA expression (or cognate empty vector) and 0.05 μg of plasmid to express the minigene. Total RNA was purified using a NucleoSpin RNA kit (Takara Bio, Inc., Shiga, Japan) including DNase treatment. Total RNA (0.5 μg) was subjected to reverse-transcription using Revertra Ace -α- (Toyobo) with a 1:1 mixture of oligo dT and random hexamers as a primer. RT-PCR was performed using Blend Taq –plus- (Toyobo) and a panel of Fw and Rv primer sets as follows: EGFP-C1-Fw and TREM2-ex4-Rv for WT(ex2-4) and NHD(ex2-4) minigenes; TREM2-ex2-Fw2 and RT-TREM2-ex4-Rv for fl-WT and fl-NHD minigenes. PCR products were resolved by electrophoresis on 2% agarose gels or e-PAGEL polyacrylamide gels (ATTO, Tokyo, Japan) and the gels were stained with ethidium bromide (Genesee Scientific Corporation, San Diego, CA, USA) or GelRed (Wako). By sampling at multiple cycles, the cycle numbers of PCR were adjusted such that the amplification was within a logarithmic phase. The gel images were captured by either a digital camera (FAS-digi, Nippon Genetics, Tokyo, Japan) or CCD camera (WSE-6200H LuminoGraph II, ATTO). Multigauge software (FUJIFILM, Tokyo, Japan) was used to quantify the splicing patterns.

### Splicing signal analysis

Splice site strength was analyzed using the website Spice Site Score Calculation (http://rulai.cshl.edu/new_alt_exon_db2/HTML/score.html) using the splice site sequence of human *TREM2* exons as inputs. A similar analysis was conducted using the Human Splice Site Finder version 3.0 (HSF3.0, http://www.umd.be/HSF3/)^44^. We also used HSF3.0 to predict the branch point sequence in *TREM2* intron 2.

### Establishment of cell lines

Flp-In-293 cells (Thermo Fisher Scientific) were transfected with either fl-WT or fl-NHD and pOG44 at a ratio of 1:9. Cells were selected after a treatment with 62.5 ng/mL of hygromycin B (Thermo Fisher Scientific). Cell clones derived from single cells were established. Integration of *TREM2* fragments into the target genomic region was confirmed by PCR using genomic DNA isolated from the established cell lines. Cells were maintained in culture medium containing 50 ng/mL of hygromycin.

### SDS-PAGE and western blot analysis

For protein expression analyses, cells were lysed by sonication in PBS containing 2% SDS. The protein concentration of the cell lysates was quantified using a Pierce BCA protein assay kit (Rockford, IL, USA). After adjusting the protein concentration, cell lysates were mixed with SDS-sample buffer and boiled. SDS-PAGE and western blot analyses were performed as previously described^45^. We used the following antibodies: anti-human TREM2 (goat polyclonal, R&D Systems, Minneapolis, MN, USA), anti-HSP60 (goat polyclonal, Santa Cruz Biotechnology, Dallas, TX, USA), and donkey anti-goat IgG-HRP (Santa Cruz Biotechnology).

### Immunofluorescence

fl-WT and fl-NHD stable cell lines were cultured in 4-well chamber slides (Nunc Lab-Tek II Chamber Slide system, Roskilde, Denmark) and transfected with 0.25 μg plasmids to express modified snRNAs. Forty-eight hours after transfection, the cells were fixed with 4% paraformaldehyde and permeabilized with PBS containing 0.1% Triton X-100 for 5 min at room temperature. After blocking with 5% skim milk for at least 30 min, the cells were incubated with anti-human TREM2 (1:200) at 4 °C overnight. Next, the cells were incubated with Alexa568-conjugated donkey anti-goat secondary antibody (1:1000; Thermo Fisher scientific) for 1 h at room temperature. After washing, the cells were treated with mounting medium with DAPI (VECTASHIELD, Vector Laboratories, Burlingame, CA, USA). Fluorescence images were captured using either a fluorescence microscope (EVOS FL Cell Imaging System, Thermo Fisher scientific) or confocal microscope (LSM710, Zeiss, Oberkochen, Germany).

### Data availability statement

The datasets generated during and/or analyzed during the current study are available from the corresponding author on reasonable request.

## Electronic supplementary material

Supplementary Information
