# Supplementary material for: Small nuclear RNA-mediated modulation of splicing reveals a therapeutic strategy for a TREM2 mutation and its post-transcriptional regulation
Source: Sci Rep. 2018 May 2;8:6937. doi: 10.1038/s41598-018-25204-2 (PMC5931963; doi:10.1038/s41598-018-25204-2)
Supplement: Supplementary file 1 — Supplementary Information [file 41598_2018_25204_MOESM1_ESM.pdf]

Supplementary Information

Small nuclear RNA-mediated modulation of splicing reveals a therapeutic strategy for a *TREM2* mutation and its post-transcriptional regulation

Motoaki Yanaizu, Kenji Sakai, Youhei Tosaki, Yoshihiro Kino, Jun-ichi Satoh.

Supplementary Table S1

Table S1 Primer sequence

Purpose	Name	Sequence (5' to 3')
TREM2 ex2-4 minigene construction	TREM2-int1-Fw	AAGGTCCCCTTGTCCTCAGCTCTTGATGGGAGGTG
	TREM2-ex5-Rv	AGGAGTCCTGGTGGCCAAGTGGCAAGTATGCAGGC
	BclI-TREM2-Fw	AAAATGATCACTGTCCGGAGCCCACAACACCACAG
	SalI-TREM2-Rv	AAAAAGTCGACCTGGCAGAGTTTGGAGCTGATACC
fl-TREM2 construction	TREM2-ex1-5'UTR-Fw	GACGAGATCTTGCACAAGGCACTCTGCTTCTGCCC
	nest-TREM2-ex5-Rv	TGTCCAATATTCAGAAGTTGTCAGGTGTTCTTACC
	NheI-TREM2-ex1-Fw	AAAAGCTAGCATGGAGCCTCTCCGGCTGCTCATCT
	NotI-TREM2-ex5-Rv	AAAAGCGGCCGCTCAACCAGTCCCTGCTTCCAGGG
TREM2 GC-opt construction	TREM2-GC-opt-Fw	GCACAGCATCTCCAGGCAAGTATATGGGTCTTTCC
	TREM2-GC-opt-Rv	GACCCATATACTTGCCTGGAGATGCTGTGCTCCAC
modified U1 snRNA construction	nest-U1-Fw	TTCATAAATATGGCTTCTCTCTGATATTAAACGGC
	nest-U1-Rv	TACGCAAAGTAAGCGTGTTATGTCTACAACCCAAC
	BglII-U1 snRNA-Fw	AAAAAGATCTGTAATCCGAAACATTCTAGTCTGCG
	BamHI-U1 snRNA-Fw	AAAAGGATCCGTAATCCGAAACATTCTAGTCTGCG
	XhoI-U1 snRNA-Rv	AAAACTCGAGCCACTGTAGGATTAACACCTAAGAC
	mut-U1-NHD-Fw	GGCACAACGTTTCAGCTGTGCCTGGCAGGGGAGAT
	mut-U1-NHD-Rv	ATCTCCCCTGCCAGGCACAGCTGAAACGTTGTGCC
	NHD-U1.4-Fw	AAAGATCTCAGCTGTGCCTGGCAGGGGAGATACCA
	NHD-U1.4-Rv	CCCTGCCAGGCACAGCTGAGATCTTTGGGCTCTGC
	U1-NHD-Fw2	CCAAAGATCTCATCTGTGCCTGGCAGGGGAGATAC
	U1-NHD-Fw3	CCAAAGATCTCATATGTGCCTGGCAGGGGAGATAC
	U1-NHD-Fw4	CCAAAGATCTCATACGTGCCTGGCAGGGGAGATAC
	U1-NHD-Fw5	CCAAAGATCTCATACTTGCCTGGCAGGGGAGATAC
modified U7 snRNA construction	BglII-Rnu7-Fw	AAAAAGATCTTAACAACATAGGAGCTGTGATTGGC
	XhoI-Rnu7-Rv	AAAACTCGAGCACATACGCGTTTCCTAGGAAACCA
	U7-TREM2-ex3-A-Fw	GGACGGAGGACTAAGCTCTCAGACTCCCCGGAGAATTTTTGGAGCAGGTTTTCTGACTTCGGTCGGAAAACCCCT
	U7-TREM2-ex3-A-Rv	TCCGGGGAGTCTGAGAGCTTAGTCCTCCGTCCTCCGTCCTCCTTGCGGAAGTGCGTCTGTAGCGAGCCAGGGAAG
	U7-TREM2-ex3-B-Fw	GGACGGAGGACTGATGCTGTGCTCCACATGGAGAATTTTTGGAGCAGGTTTTCTGACTTCGGTCGGAAAACCCCT
	U7-TREM2-ex3-B-Rv	TCCATGTGGAGCACAGCATCAGTCCTCCGTCCTCCGTCCTCCTTGCGGAAGTGCGTCTGTAGCGAGCCAGGGAAG
	U7-TREM2-ex3-C-Fw	GGACGGAGGACTCCAGCATCCCGGTGATCCAAGAATTTTTGGAGCAGGTTTTCTGACTTCGGTCGGAAAACCCCT
	U7-TREM2-ex3-C-Rv	TTGGATCACCGGGATGCTGGAGTCCTCCGTCCTCCGTCCTCCTTGCGGAAGTGCGTCTGTAGCGAGCCAGGGAAG
	U7-TREM2-ex3-D-Fw	GGACGGAGGACTGGAACCAGAGATCTCCAGCAGAATTTTTGGAGCAGGTTTTCTGACTTCGGTCGGAAAACCCCT
	U7-TREM2-ex3-D-Rv	TGCTGGAGATCTCTGGTTCCAGTCCTCCGTCCTCCGTCCTCCTTGCGGAAGTGCGTCTGTAGCGAGCCAGGGAAG
	U7-TREM2-ex3-E-Fw	GGACGGAGGACTCCACATGGGCATCCTCGAAGCTCTCAGAATTTTTGGAGCAGGTTTTCTGACTTCGGTCGGAAAACCCCT
	U7-TREM2-ex3-E-Rv	TGAGAGCTTCGAGGATGCCCATGTGGAGTCCTCCGTCCTCCGTCCTCCTTGCGGAAGTGCGTCTGTAGCGAGCCAGGGAAG
U7 TREM2-ex3-skip construction	U7-TREM2-ex3-skip-Fw	AGACCCATCGCTGTACCTGGAGGCCATGAGCCTCCAGCCCCTTCCAGAATTTTTGGAGCAGGTTTTCTGA
	U7-TREM2-ex3-skip-Rv	GGAAGGGGCTGGAGGCTCATGGCCTCCAGGTACAGCGATGGGTCTTTGCGGAAGTGCGTCTGTAGCGAGC
TREM2 ex2-4 splicing assay	EGFP-C1-Fw	CATGGTCCTGCTGGAGTTCGTG
	TREM2-ex4-Rv	CTGGCAGAGTTTGGAGCTGATACCCTGGGTCATGG
fl-TREM2 splicing assay	TREM2-ex2-Fw2	AGGTGTCTTGCCCCTATGACTCCATGAAGCAC
	RT-TREM2-ex4-Rv	AGGAGGAGAAGGATGGAAGT
RNAi	siUPF1-404-S (siRNA)	gcaagaagugguucugcaaTT (RNA 19wer + TT)
	siUPF1-404-AS (siRNA)	uugcagaaccacuucuugcTT (RNA 19wer + TT)
NMD analysis	SRSF6-ex2-Fw	GTACGGCTTCGTGGAGTTCGAGGACTCCCGCGAC
	SRSF6-ex4-Rv	CTTTAAATCTTGCCAACTGCACCGACTAGAAAGAT
	hUPF1-Fw	TCAACCACTCCCAGGTTTATGCCGTGAAGACTGTG
	hUPF1-Rv	AAAATGGAGCGGAACTGCATCTTGGCCAGCCTCGG
	hGAPDH-Fw	GAACATCATCCCTGCCTCTACTGGCGCTGCCAAGG
	hGAPDH-Rv	CGTTGTCATACCAGGAAATGAGCTTGACAAAGTGG

Supplementary Figure S1

A

U7 WT mouse AAGTGTTACAGCTCTTTTAGAATTTGTCTAGCAGGTTTTCTGACTTCGGTCGGAAAACCCCT

U7 smOPT AAGTGTTACAGCTCTTTTAGAATTTTTGGAGCAGGTTTTCTGACTTCGGTCGGAAAACCCCT

SmOPT

B

U7 ESE-TREM2-ex3-A (U7A)

AAGGAGGACGGAGGACGGAGGACTAAGCTCTCAGACTCCCCGGAGAATTTTTGGAGCAGGTTTTCTGACTTCGGTCGGAAAACCCCT

Antisense: exon3-A

U7 ESE-TREM2-ex3-B (U7B)

AAGGAGGACGGAGGACGGAGGACTGATGCTGTGCTCCACATGGAGAATTTTTGGAGCAGGTTTTCTGACTTCGGTCGGAAAACCCCT

Antisense: exon3-B

U7 ESE-TREM2-ex3-C (U7C)

AAGGAGGACGGAGGACGGAGGACTCCAGCATCCCGGTGATCCAAGAATTTTTGGAGCAGGTTTTCTGACTTCGGTCGGAAAACCCCT

Antisense: exon3-C

U7 ESE-TREM2-ex3-D (U7D)

AAGGAGGACGGAGGACGGAGGACTGGAACCAGAGATCTCCAGCAGAATTTTTGGAGCAGGTTTTCTGACTTCGGTCGGAAAACCCCT

Antisense: exon3-D

U7 ESE-TREM2-ex3-E (U7E)

AAGGAGGACGGAGGACGGAGGACTCCACATGGGCATCCTCGAAGCTCTCAGAATTTTTGGAGCAGGTTTTCTGACTTCGGTCGGAAAACCCCT

Antisense: exon3-E

C

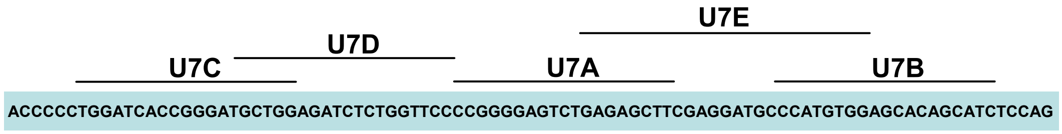

TREM2 exon 3

Figure S1. Sequences of modified U7 constructs.

(A) DNA sequences of wild-type (unmodified) U7snRNA and its variants. An AATTTGTCT motif (boxed) is a binding motif for LSm proteins. In the engineered U7 snRNAs, this sequence was replaced with the SmOPT sequence (AATTTTTGG, indicated in red) which is recognized by splicing-associated LSm proteins. **(B)** DNA sequences of modified U7 snRNA constructs (U7A-U7E). All contain an exonic splicing enhancer (ESE, blue letters), antisense oligonucleotides of *TREM2* exon 3 (underlined), and SmOPT sequence (red letters). The ESE is recognized by SRSF1 and thought to enhance exon inclusion. **(C)** The location of target sites on *TREM2* exon 3 recognized by the antisense oligonucleotides in the modified U7 constructs.

Supplementary Figure S2

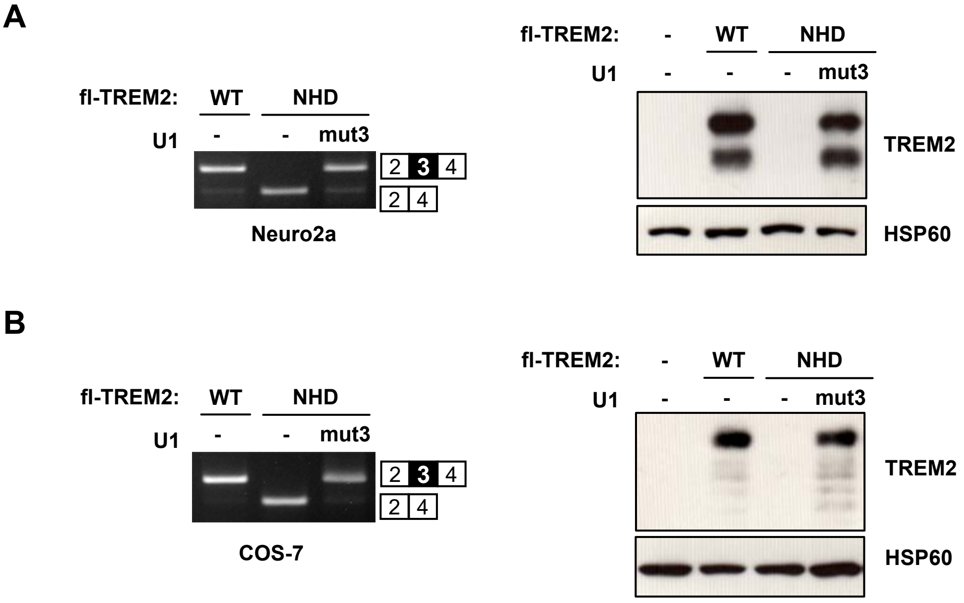

Figure S2. Effect of U1 and U7 constructs in different cell types.

(A) *TREM2* minigenes were transfected into Neuro2a cells together with modified U1 and U7 constructs. The splicing patterns of the *TREM2* minigene were detected by RT-PCR. **(B)** Splicing assay of *TREM2* minigenes in COS-7 cells.

Supplementary Figure S3

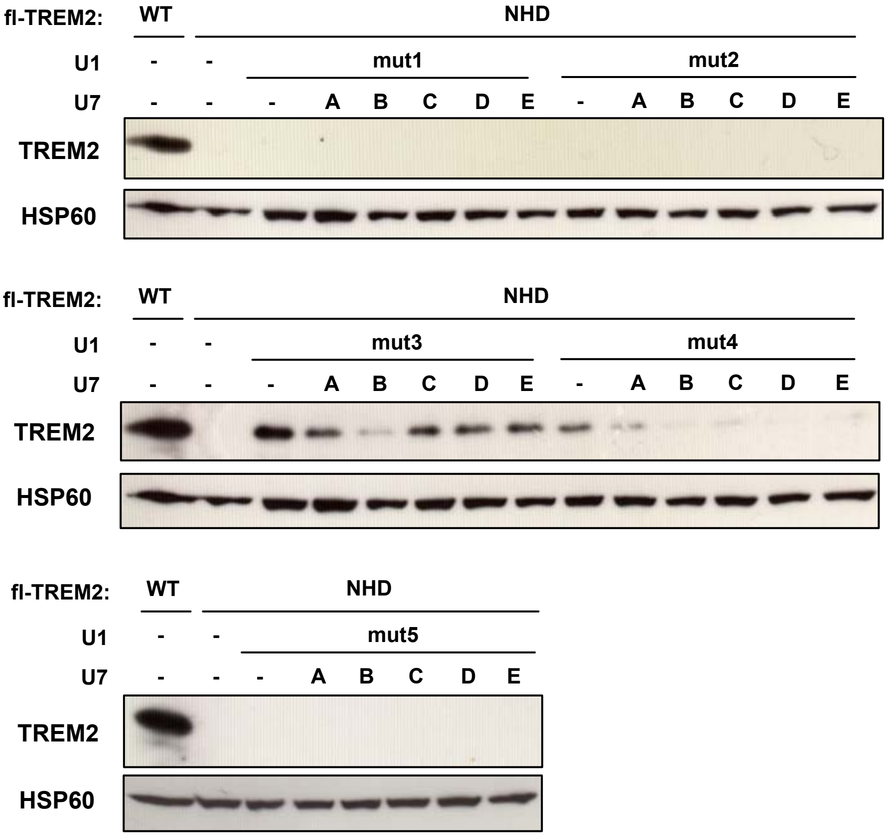

Figure S3. Effect of U1 and U7 constructs in combinations.

Modified U1 and U7 constructs were transfected into fl-TREM2(NHD) cells in the combinations indicated and the splicing pattern was analyzed by RT-PCR. TREM2 protein expression by U1mut3 or U1mut4 was not enhanced by co-expression of U7 constructs.

Supplementary Figure S4

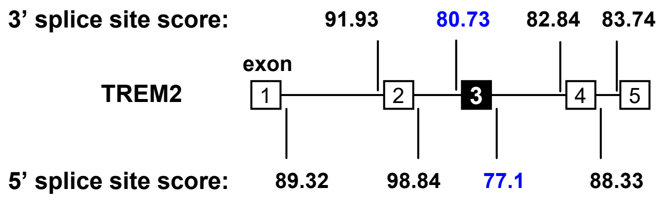

Figure S4. Splice site score estimation of *TREM2* using Human Splice Finder 3.0 (HSF3.0).
Both 5' and 3' splice sites of human *TREM2* were analyzed using HSF3.0. The splice sites flanking exon 3 were the weakest among the *TREM2* splice sites. Note that exon 4 is known to be an alternative exon.

Supplementary Figure S5

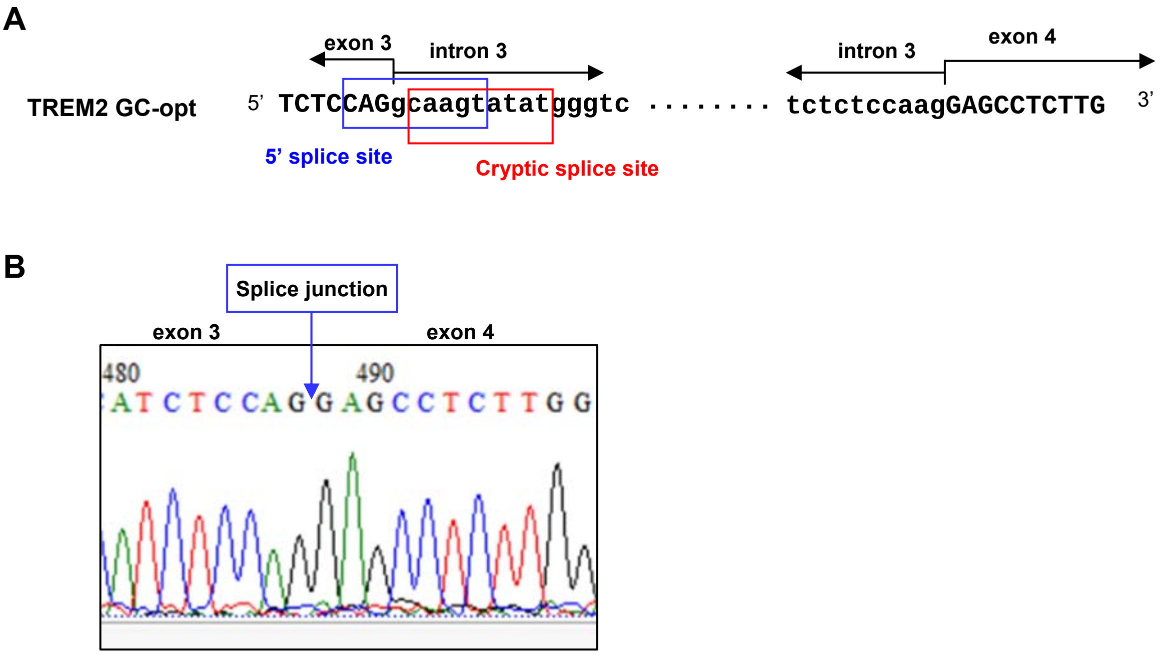

Figure S5. Cryptic splice site of the GC-opt minigene is not used for splicing.

(A) Sequences around the 5' splice site of exon 3 and 3' splice of exon 4 in the GC-opt minigene. Red box indicates a cryptic splice site generated by substitution of GC-opt, which is located four nucleotides downstream of the original 5' splice site. **(B)** Sequence of the amplicon of the GC-opt minigene. The splice junction between exons 3 and 4 is shown. The original 5' splice site, but not the cryptic site, was chosen for splicing.

Supplementary Figure S6

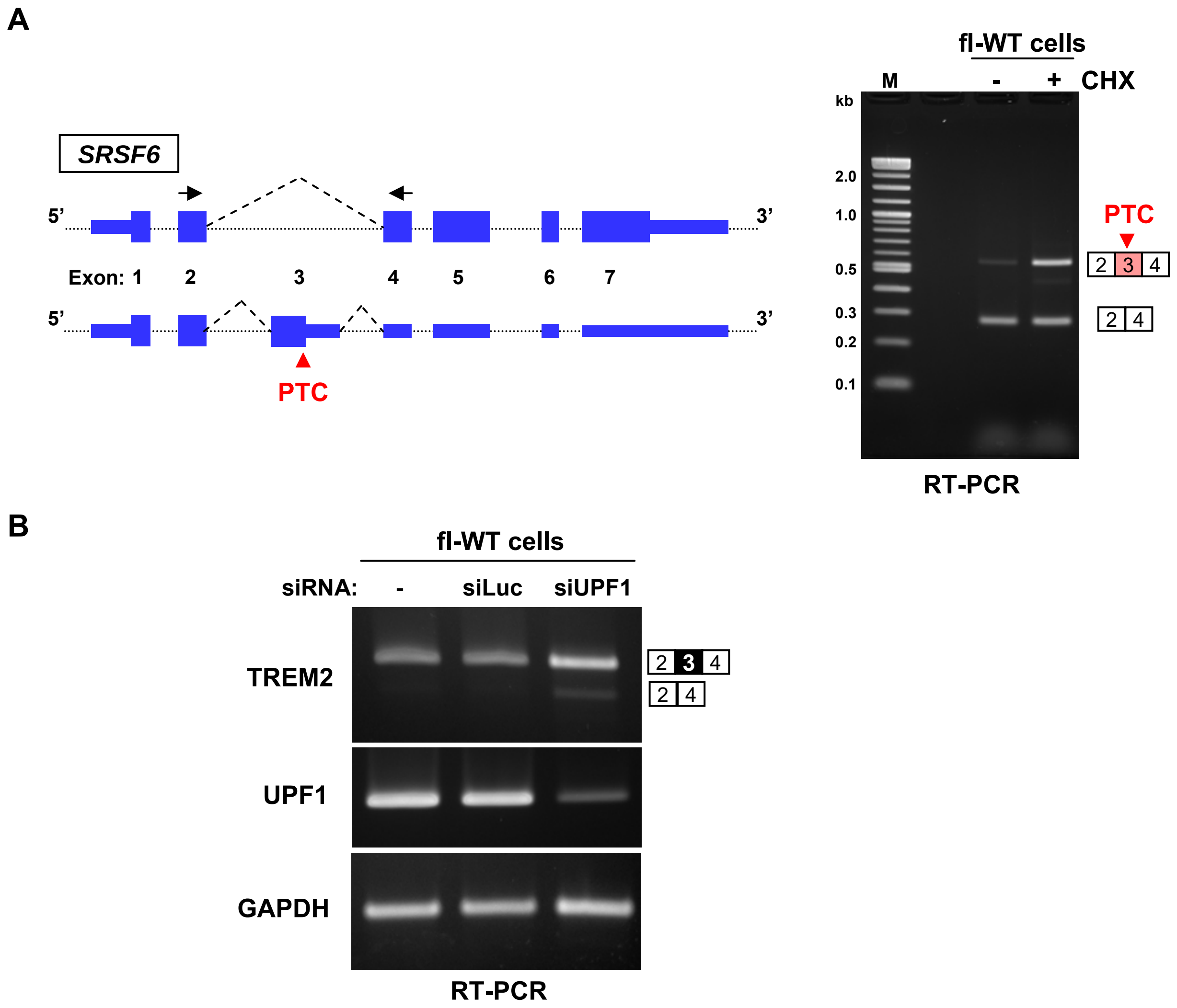

Figure S6. NMD is involved in suppressing *TREM2* transcripts lacking exon 3

(A) Cycloheximide treatment increased an NMD-sensitive isoform of *SRSF6*. Left: schematic diagram of the exon structure of *SRSF6*. Exon 3 is an alternative exon containing a premature termination codon (PTC, red arrow head). Right: splicing assay result of *SRSF6* using the samples analyzed in Fig. 5B. CHX treatment increased the NMD-sensitive isoform of *SRSF6*. **(B)** Knockdown of *UPF1* increased the amount of exon3-lacking isoform of TREM2. Endogenous UPF1 was depleted by RNAi-mediated knockdown using siRNA targeting *UPF1* (siUPF1) but not luciferase (siLuc) in fl-WT cells. RT-PCR results of *TREM2*, *UPF1*, and *GAPDH* are shown.

Supplementary Figure S7

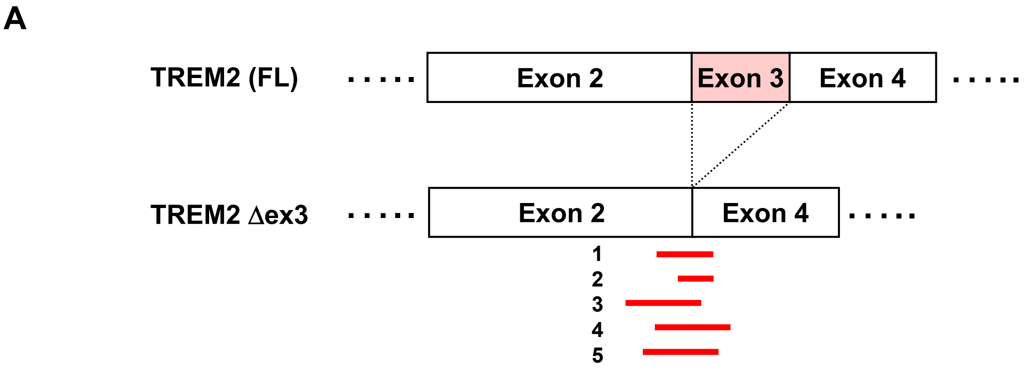

#	GEO accession	SRA ID	Cell type	Accession
1	GSM2285371	SRX2035386	iPS-derived microglia-like cell	SRA:SRR4044513.29135611.2
2	GSM1697106	SRX1040867	THP-1	SRA:SRR2042590.17737324.1
3	GSM2599710	SRX2785186	THP-1	SRA:SRR5511207.6999748.1
4	GSM2551706	SRX2676718	iPS-derived microglia	SRA:SRR5381591.37824307.1
5	GSM2803868	SRX3242697	Primary macrophage	SRA:SRR6130321.7263708.1

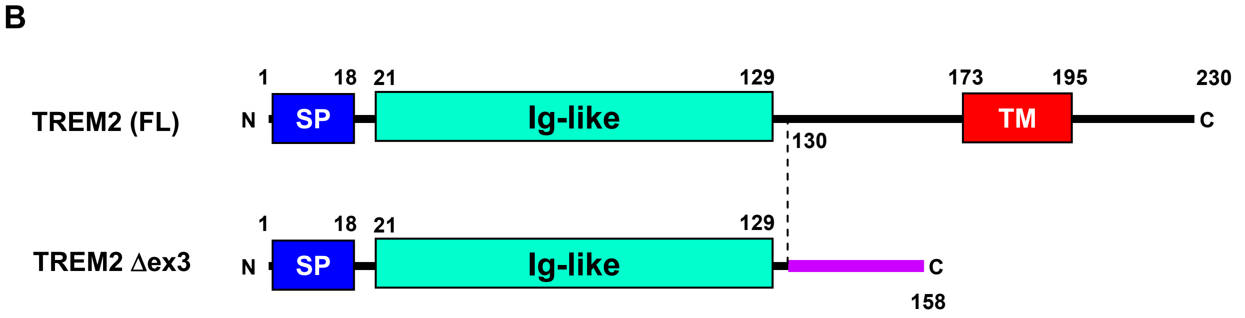

Figure S7. Supporting data for exon 3 skipping of *TREM2* and the predicted structure of *TREM2* Δex3.

(A) RNA-seq data from several independent data sets. Red horizontal lines indicate RNA-seq reads overlapping with the junction of exons 2 and 4. **(B)** Schematic diagram of both full-length and Δex3 TREM2 isoforms. SP: signal peptide. Ig-like: immunoglobulin-like domain. TM: transmembrane domain. The C-terminus of TREM2 Δex3 is distinct from the full-length isoform.

Supplementary Figure S8

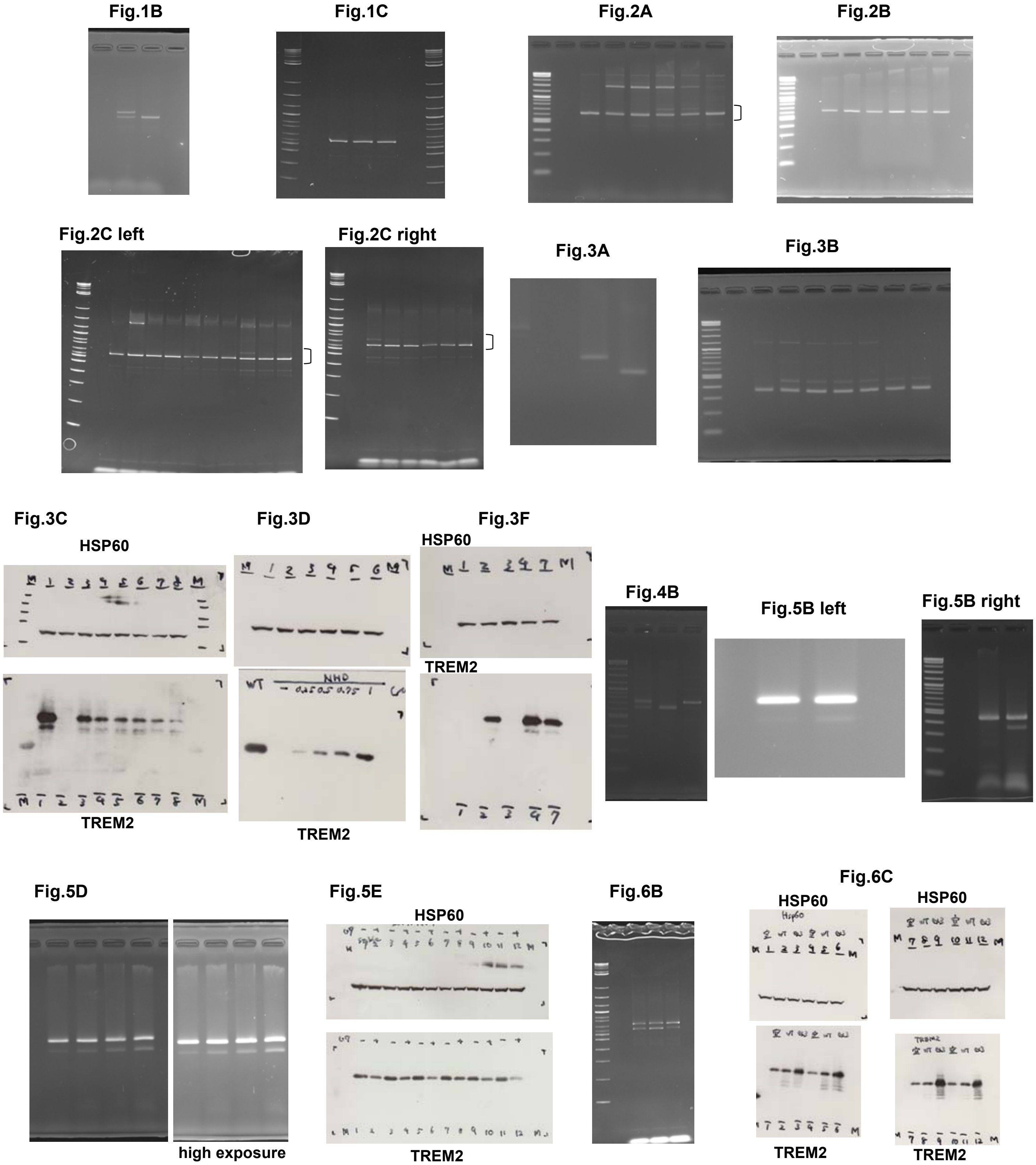

Figure S8. Original images obtained from western blot and RT-PCR analyses.

For western blot analyses of TREM2 and HSP60, transferred membranes were cut at ~50 kDa, and the upper and lower parts were stained with anti-HSP60 and anti-TREM2, respectively.
